# Operation of Three-Stage Process of Lithium Recovery from Geothermal Brine: Simulation

**DOI:** 10.3390/membranes11030175

**Published:** 2021-02-28

**Authors:** Denis Kalmykov, Sergey Makaev, Georgy Golubev, Ilia Eremeev, Vladimir Vasilevsky, Jianfeng Song, Tao He, Alexey Volkov

**Affiliations:** 1 A.V.Topchiev Institute of Petrochemical Synthesis RAS, 29 Leninsky prospekt, 119991 Moscow, Russia; denis.kalmykov@ips.ac.ru (D.K.); makaev@ips.ac.ru (S.M.); golubevgs@ips.ac.ru (G.G.); eremeevis@ips.ac.ru (I.E.); vasilevskii@ips.ac.ru (V.V.); 2 National Research Nuclear University Moscow Engineering Physics Institute MEPhI, 115409 Moscow, Russia; 3 Shanghai Advanced Research Institute, Chinese Academy of Sciences, Shanghai 201210, China; songjf@sari.ac.cn (J.S.); het@sari.ac.cn (T.H.)

**Keywords:** lithium, geothermal brine, membrane distillation, porous condenser, membrane extraction, PHREEQC, Simulink/MATLAB

## Abstract

Lithium-rich geothermal waters are considered as an alternative source, and further concentration of lithium is required for its effective recovery. In this work, we have simulated a three-stage lithium recovery process including the brine softening by precipitation Ca^2+^/Mg^2+^ cations with sodium carbonate (calculated in PHREEQC), followed by an integrated system consisting of membrane distillation unit (water evaporation), crystallizer (NaCl precipitation), and membrane extraction (Li^+^ recovery), which was simulated in Simulink/MATLAB. It was shown that the deterioration of membrane performance in time due to scaling/fouling plays a critical role in the performance of the system resulting in the dramatic increase of the replaced membrane modules by a factor of 5. Low cost membranes are required. The process simulation based on the experimental and literature data on the high salinity solutions with the membrane distillation revealed that the specific productivity can be achieved in the range of 9.9–880 g (Li^+^) per square meter of membranes in the module used before its replacement. The increase of energy efficiency is needed. The mass-flow-rate of saline solution circulated to the crystallizer was set at its almost minimum value as 6.5 kg/min to enable its successful operation at the given parameters of the membrane distillation unit. In other words, the operation of the integrated system having 140 kg of saline solution in the loop and a membrane module of 2.5 m^2^ for concentration of lithium presence from 0.11 up to 2.3 g/kg would be associated with the circulation of about of 259 tons of saline solution per month between the distillation unit (60 °C) and the crystallizer (15 °C) to yield of up to 1.4 kg of lithium ions. The comprehensive summary and discussion are presented in the conclusions section.

## 1. Introduction

The development of new technologies over the past decade has been associated with a sharp increase in lithium consumption, which is evident by a tenfold increase in lithium production from 1995 to 2019, reaching about 80,000 tons per year [1]. This is mainly due to the growing demand for lithium-ion batteries (about 65% of all lithium mined in 2019) for electric vehicles, energy accumulation, and electronic equipment [2]. The electrochemical potential (3.045 V) and density (534 kg/m^3^) of lithium [2,3] give the batteries the highest specific and volumetric energy density (more than 160 W∙h/kg), which is more than twice the nickel-cadmium (~50W∙h/kg) and nickel-metal hydride (~70 W∙h/kg) batteries [4]. In addition to batteries, lithium is widely used in the ceramics and glass industries (~18%), the production of lubricants (~5%), polymer and chemical technologies, metallurgy, air purification, and other areas such as medicine, hydrogen, and nuclear energy [1,5,6,7,8,9].

Due to its chemical activity, lithium can be found in nature only in the form of salts presented in more than 150 different minerals, solid ores (spodumene, petalite, and lepidolite), and salt brines (salt lakes and geothermal brines) [10]. Today, the total lithium reserves worldwide are estimated as 80 million tons [1], and more than 60% of lithium is presented in the brines [1,11,12,13]. Despite the fact that about 83% of lithium is produced today from the brines due to its lower production cost [5], the ores remain the main sources of lithium in some countries like Australia [1]. The recovery of lithium from a different kind of natural brines such as geothermal brine is complicated by high water salinity 200–500 g/L and the presence of hardness ions, especially magnesium ions [13,14]. Li^+^ and Mg^2+^ ions have very similar size of 0.076 and 0.075 nm, respectively, which requires additional efforts and processing steps in the case of a high Mg^2+^/Li^+^ ratio [15,16]. Among different methods of lithium recovery (e.g., extraction, crystallization, precipitation, ion-exchange adsorption, electrodialysis), extraction and ionic adsorption are most widely used due to their high selectivity [10,17,18,19,20,21,22,23,24]. The membrane-based extraction (ME) can be considered a novel and promising method due to its modularity and the absence of direct contact of aqueous and organic phases that prevents the extractant loss and negative impact on the environment [22,23,24]. High separation characteristics (productivity and selectivity) were demonstrated with ethylene–vinyl alcohol (EVAL) membranes in the membrane extraction [23]. The membrane prepared from 30% EVAL showed a Li^+^ flux of 6.8 g/m^2^∙h at a Li^+^ feed concentration of 0.7 g/L. However, these membranes possess limited stability under operation conditions (extractant regeneration by strong hydrochloric acid). To overcome this problem, it was recently proposed to use acid-resistance membranes based on poly(ether-ether-ketone) (PEEK) for solvent regeneration; PEEK membranes demonstrated stable performance during the long-term operation of 500 h [24]. It should be noted that the membrane contactor demonstrates effective operation with a high mass transfer rate when lithium concentration in the feed solution is at the preferable level (2 g/L or higher). As can be seen from Table 1, lithium concentration in the prospective geothermal brines in Russian Federation varies from 0.01 up to 0.5 g/L [25,26], which requires the prior concentration of the initial geothermal brine by a factor of 4–200 in order to achieve the required lithium content. However, such treatment is associated with scaling and salt deposition due to high initial salinity and the pronounced presence of calcium and magnesium in some cases, which would result in the drop of membrane productivity, deterioration of heat and mass transfer, and the increasing of hydraulic resistance [27].

The scaling phenomena can be mitigated by the addition of special reagents like slaked lime (Ca(OH)_2_) or soda (Na_2_CO_3_), resulting in the precipitation of hardness ions in the form of insoluble carbonate compounds (CaCO_3_, MgCO_3_, CaMg(CO_3_)_2_) [28,29]. Lime removes carbonate salts, while soda removes non-carbonate salts [30]. However, this method requires a high amount of reagents, which is associated with the introduction of additional ions to the brine solution [31].

In the 2010s, a new method called membrane-assisted crystallization was proposed for simultaneous water production using membrane technology and recovery of dissolved compounds in the solid form by its crystallization from saturated solutions [32]. This approach is compelling because of its compactness, lesser metal consumption, and greater flexibility in the process operation, and it was already utilized for crystallization of inorganic salts [33,34] and organic compounds [35] using pressure-driven processes of solution concentration (reverse osmosis, nanofiltration) as well as evaporation processes (pervaporation, membrane distillation). The membrane distillation (MD) can be considered one of the effective approaches for the concentration of different kinds of brines with moderate or high salinity [36]. In this thermally-driven process, the membrane acts as a non-selective barrier that provides high fluxes of water vapors through the pores of the membrane and high selectivity since the saline water as the non-wetting liquid for hydrophobic membrane remains in the feed compartment [37,38]. In contrast to pervaporation, reverse osmosis or distillation processes, the membrane distillation can be effectively operated at the atmospheric pressure by utilizing low-grade heat sources [38,39,40,41,42,43,44,45]. A variety of MD processes can provide different configurations of hot (feed) and cold (permeate) streams, which are usually positioned within the membrane module on a distance no greater than few millimeters. Recently, a new construction of air gap MD module was proposed [43,45,46,47] and patented [48]; this concept allows the intensification of the membrane distillation process. The key engineering element of this construction is a porous condensing surface (porous condenser) for condensation of water (permeate) vapors, which allows to scale up the process to the industrial level, simplify the construction, reduce the dimensions, and use the membrane module with any spatial orientation without the loss of efficiency. Using the simulation software Simulink (MATLAB), a membrane distillation process with a porous condenser using solar energy collectors for desalination of seawater was modeled. Simulations had shown the advantage of using solar panels, which reduced energy costs up to 61% in the process of desalination of seawater [46]. Previously, the use of membrane crystallization together with membrane distillation with a porous condenser has not been investigated, not to mention the extraction of lithium from geothermal brines with high total salt content. In addition, it is of interest to carry out a full cycle of extraction of lithium from geothermal brines, from pre-preparation of geothermal brine to the production of commercial lithium chloride using promising processes such as membrane crystallization and membrane extraction. Before the implementation of the integrated technologies, the entire process should be modeled to check the feasibility of carrying out the proposed processes. Process simulation has become an established and widely used tool for performance calculation, design, and optimization of process parameters.

**Figure 1:**
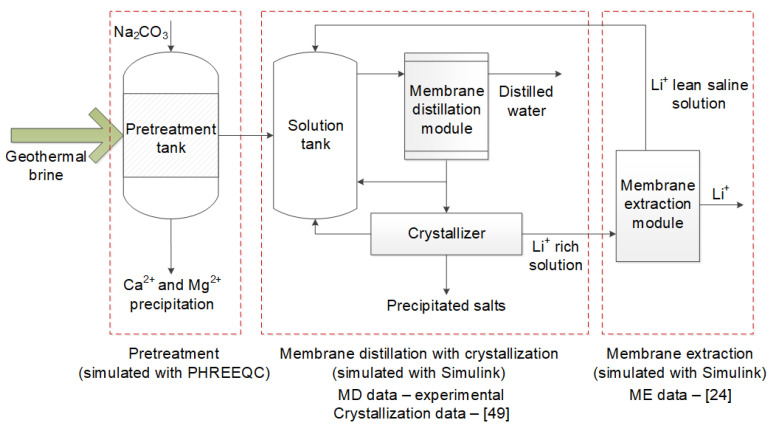
Principal scheme of three-stage process for lithium recovery from geothermal brine.

Therefore, the goal of this work was to simulate the operation of the lithium recovery process from geothermal brines with the primary focus on the performance of the membrane distillation unit used for salt concentration. The process consisted of three different stages as shown in Figure 1: (i) removal of hardness ions from geothermal brine by leaching with sodium carbonate, (ii) concentration of a pre-treated solution by using air–gap membrane distillation equipped with membrane condenser (AGMD-MC), and (iii) membrane extraction of lithium ions. The composition of the model solution after sodium carbonate pre-treatment used as the feed for the stage of membrane distillation with crystallization was calculated with the PHREEQC program, and the performance of the last two stages was modeled using Simulink program. Simulink is an add-on to MATLAB and is a graphical programming environment for modeling the behavior of the system over time (dynamic modeling). Unlike classical programming languages and MATLAB itself, Simulink has a relatively low entry threshold because it does not require writing extensive program code; instead, graphic blocks are used to describe mathematical expressions [46].

## 2. Materials and Methods

### 2.1. Experimental Study of Air–Gap Membrane Distillation with Porous Condenser

The laboratory set-up of membrane distillation with a porous condenser used for concentration of sodium chloride aqueous solution was described elsewhere in [43,45,46,47]. The feed solution with an initial concentration 10 wt.% of NaCl was circulated in the membrane distillation module at a temperature of 60 or 80 °C at a flow-rate of 0.012 m/s. The temperature of the coolant (distilled water) in the porous membrane condenser was kept at 20 °C (the flow-rate 0.3 L/min). The MD module with an active surface area of 146 cm^2^ was equipped with a commercial microfiltration membrane MFFK-1 (Vladipor Scientific and Technical Center, Vladimir, Russia); porous condenser with a thickness of 200 μm and porosity of 30% was made of sintered stainless steel (OOO VMZ-Techno, Moscow, Russia,). MFFK-1 (0.15 μm pore size, 85 % total porosity) consisted of a porous top-layer based on fluoroplastic F42L (a copolymer of tetrafluoroethylene and vinylidene fluoride) and non-woven support with. The air gap between MFFK-1 and porous condenser was set at 3 mm.

### 2.2. Simulation of the Pretreatment Stage with PHREEQC

The geothermal brine from the Udachnaya pipe (Sakha Republic, Russian Federation) with Li^+^ content of 0.14 g/L (see Table 1) was considered in this work. To prevent membrane scaling, calcium (11.2 g/L) and magnesium (65.5 g/L) ions were precipitated from the geothermal brine by the treatment with sodium carbonate. The composition of resulted solution upon addition of a different amount of sodium carbonate was calculated by the PHREEQC program with Pitzer database. PHREEQC codes used for conversion of the concentration from g/L to molality and for simulation of CaCO_3_ and MgCO_3_ precipitation by Na_2_CO_3_ are listed in Supplementary S1 and S2.

### 2.3. Simulation of Membrane Distillation/Crystalization and Lithium Extraction with Simulink/MATLAB

**Figure 2:**
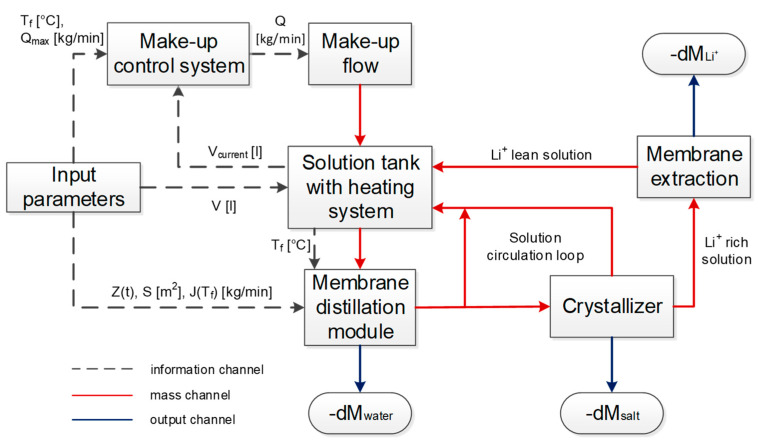
The schematic flow-sheet diagram of the system.

The integrated operation of membrane distillation, salt crystallization, and lithium extraction processes were simulated with several subsystems in Simulink/MATLAB. The integrations and connections between different subsystems (units) are schematically represented in Figure 2. The salt solution with a composition presented in Table 2 and having the density of 1210 kg/m^3^ was fed to “solution tank with the heating system” to be heated up to 60 °C, and this temperature was kept by the system within ±1 °C. The hot saline solution went to the “membrane distillation module” unit, where the part of the water was evaporated, and then part of the solution (~6 kg/h) was fed to the “crystallizer” unit, where the salt solution was cooled down to 20 °C and corresponded amount of salt (NaCl) was precipitated. The remained hot solution was recycled back from “membrane distillation module” to “solution tank with the heating system.” The “crystallizer” maintaining 90 kg of the solution was considered as black-box assuming that the excess of salts above the saturation concentration at 15 °C was precipitated. The crystallization time of 10 min was taken from the calculation of NaCl crystallization kinetics in [49,50]. The saline solution after the crystallizer was recycled back to the “solution tank with the heating system”. The main target of the operation of the “membrane distillation module” was to concentrate Li^+^ content to the required level sufficient for its effective recovery by membrane extraction such as 0.7 g/L [18,23,24]. The concentration factor (CF) of the initial saline solution of 5, 20, and 50 were studied, and once Li^+^ content reached the required level of 0.6, 2.3 or 6.0 g/kg, respectively, the part of the solution stream from the “crystallizer” was fed to “membrane extraction module”, where lithium ions were extracted (replaced by sodium ions) with the efficiency of 90%. Then, the remained solution was recycled back to the “solution tank with heating system” (see Figure 2). The additional simulations were also carried out with the efficiency of lithium extraction of 50 and 70%; in all cases, “membrane extraction module” was considered as black-box. The total amount of solution in the system was 140 kg, and the make-up flow of fresh salt solution with the composition listed in Table 2 to “solution tank with heating system” was set as about 0.2 kg/min and varied to maintain the mass-balance of major components with the respect of evaporated water and crystallized salts.

**Figure 4:**
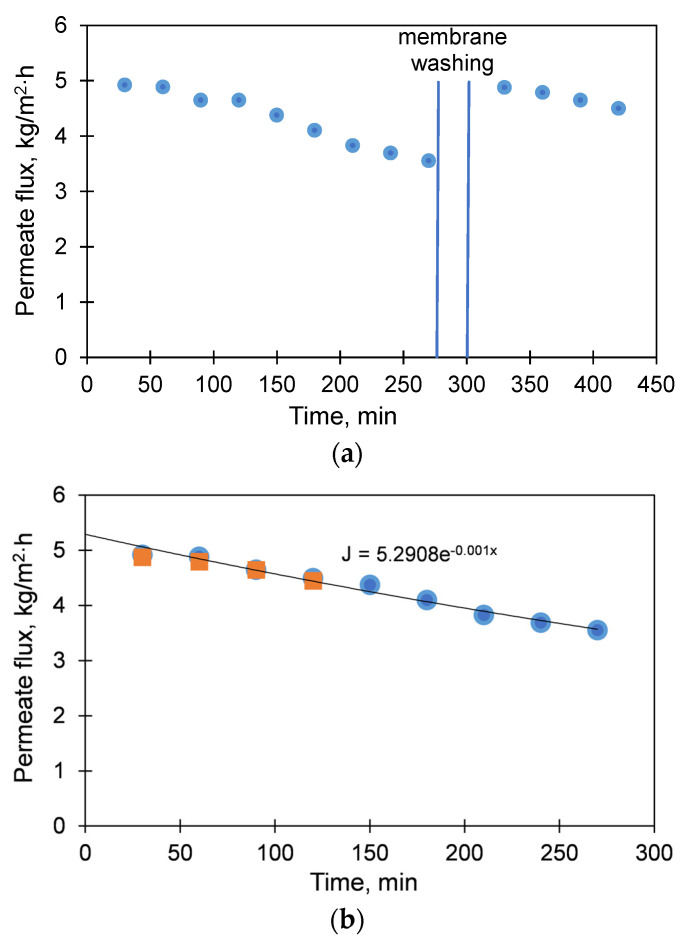
Experimental permeate fluxes in AGMD-MC module: (**a**) decrease of permeate flux before (blue dots) and after (orange squares) membrane washing; (**b**) exponential approximation of the permeate flux, using data obtained before (blue dots) and after (orange squares) membrane washing. Initial feed concentration 10 wt.% NaCl, hot circuit temperature 60 °C, cold circuit temperature 20 °C, d = 3 mm.

**Figure 5:**
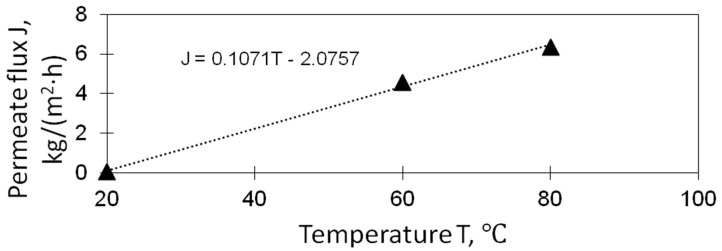
Experimental data of AGMD-MC module performance as a function of feed temperature.

As an example, Figure 3 shows Simulink code for the “membrane distillation module” subsystem, which calculated the amount of solvent (water) evaporated from the salt solution based on a number of parameters. Other subsystems are presented in the supplementary materials (Figures S1–S11). The amount of water *m* evaporated from saline solution in time [kg/min] can be described as follows:

(1) $$m=J\left(T_{f}\right)\cdot S\cdot K\cdot Z\left(t\right)\cdot 1/60$$

where *J*(*T_f_*) is the polynomial equation that defines the flux of distilled water as a function of the feed temperature; *T_f_* is the feed temperature [°C]; *S* is the active surface area of one membrane module [m^2^]; *K* is the number of membrane modules, *Z*(*t*) is the parameter describing the deterioration of membrane performance in time due to scaling/fouling. During the modeling, the temperature of the solution was varied to about 60 °C, but the temperature of the coolant/permeate was kept constant and equal to 20 °C. The polynomial equation *J*(*T_f_*) was determined from the experimental data and was taken for simulations as *J*(*T_f_*) = 0.1071 − *T_f_*-2.0757 (see Figure 5). The parameter *Z*(*t*) was determined from the experimental data as *Z*(*t*) = 5.2908∙e^−0.001∙*t*^ (see Figure 4b) used to describe the flux decline within the single operation of the membrane module during 4.5, 10, or 20 h before the membrane washing step (0.5 h). The recovery ratio of membrane performance after washing was set as 99%; in addition, 95 and 97% were also considered in this work. Once the performance of the membrane module after washing reached 50, 40, or 30% from the initial one, the membrane module was replaced by the new one. The inner working of the system was modeled using a well-known principle of proportional-integral-derivative (PID) control. Simulink performs calculations based on the input signals in every step, which avoids the need to pre-define the mathematical description for the long-term performance of the whole system.

## 3. Results and Discussion

### 3.1. AGMD-MC Experiments

Figure 4a shows typical experimental permeate fluxes and a decrease of the membrane performance over time due to reversible and irreversible fouling (hot circuit temperature 60 °C). The initial feed concentration in our experiments was 10 wt. % of NaCl, and it became saturated after ~60 min. The module was flushed for 30 min after 270 min of the experiment (Figure 4a). The recovery ratio was similar for both experiments and amounted to ~99%. The performance drop was approximated exponentially, and the resulting equations were used in Simulink (Figure 4b). The same experiments were carried out with hot circuit temperature 80 °C, and the results were similar, only with higher fluxes; this resulted in a decrease in time before the flushing (120 min instead of 270 min), but the flushing duration was intentionally the same (30 min) because we need to flush a similar amount of foulants. Performance data at different temperatures were approximated linearly using the first experimental points and taking into account zero flow when the temperatures of the hot and the cold parts of the module are equal (20 °C, Figure 5). These dependencies were used for further simulation in Simulink.

Fouling in membrane distillation with hydrophobic membranes can mainly occur due to the deposition of precipitated salt crystals and organic substances. Salt deposition can be considered as an almost reversible process because salts can be washed off with either water or dilute acid solutions (for carbonates). Deposition of organic compounds is an almost irreversible process that reduces membrane hydrophobicity by up to 70%, according to [51]. The solubility of NaCl increases from ~26.3 to ~27.3 wt. %, i.e., for only 1 wt. %, while heating from 10 to 75 °C. This greatly complicates the crystallization processes by cooling, and it is probably the main reason for very limited data on NaCl saturated solutions published in the literature. Table 3 presents the data on the performance drop in the membrane distillation with the crystallization of NaCl solutions. Most of the works either do not provide information about the long-term operation of the membrane module or do not indicate the parameters for the performance drop. Therefore, the flux drop in one run (ratio of fluxes at the beginning and the end of the run) and recovery ratio (ratio of the fluxes in the first and second run) were estimated in this work if these data were missed in the corresponded articles. As can be seen, the flux drop in one run and the recovery ratio are varied in a quite wide range, which is subject to operation conditions, membrane nature, and system configuration including the crystallization step. In this work, we used our experimental data for further simulation of membrane performance.

### 3.2. Modeling of Geothermal Brine Pretreatment

Besides the prevention of membrane scaling, calcium and magnesium ions should be removed for a number of reasons. Firstly, according to [62], magnesium ions strongly interfere with the efficiency of lithium extraction; secondly, removing calcium and magnesium reduces the mass of the solution and not only reduces the energy required for heating but also increases lithium concentration; thirdly, the lower water content in the solution leads to a lower partial vapor pressure, which affects the performance of membrane distillation modules at the same temperature. Multicomponent water–salt systems at different conditions are of high interest for geochemistry and can be described using a number of models. The classical Debye–Hückel model is correct for very dilute solutions with an ionic strength I_m_ < 0.1, but concentrated solutions require more difficult modeling. One of the most popular programs for aqueous geochemical calculations is PHREEQC. It has several built-in databases, and the two most popular are based on the Pitzer model and Specific Ion Interaction Theory (SIT) (named pitzer.dat and sit.dat). The SIT model has larger component coverage, but the Pitzer model is self-consistent and arguably the most accurate [63]. Preliminary leaching with soda ash (Na_2_CO_3_) was modeled using the PHREEQC program before the solution was fed into the membrane distillation–crystallization system. Firstly, PHREEQC was used to calculate the density of the model geothermal brine (1244.7 kg/m^3^ due to the high content of salts) and then the molalities and mass fractions of the components (Table 4).

**Figure 6:**
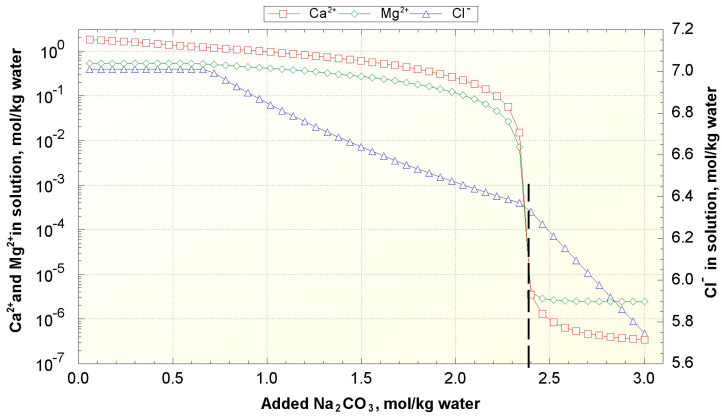
Change of Ca^2+^, Mg^2+^, and Cl^−^ molalities in geothermal brine with the addition of Na_2_CO_3_ (calculation with PHREEQC).

Next, it was used to calculate the change in the concentration of Mg^2+^, Ca^2+,^ and Cl^−^ in solution with the addition of soda ash (Figure 6). It was presumed that the system was exposed to the atmosphere; therefore, CO_2_ partial pressure was equal to the carbon dioxide content in the atmosphere (4 × 10^−4^ bar, fugacity 1). It can be seen from Figure 6 that, with the addition of 2.3–2.4 mol/kg Na_2_CO_3_ (i.e., the sum of Mg^2+^ and Ca^2+^ molalities in the initial solution, Table 2), Mg^2+^ and Ca^2+^ concentration sharply declines; further leaching does not lead to big change and is not reasonable. Therefore, the amount of added Na_2_CO_3_ was equal to the sum of Mg^2+^ and Ca^2+^ molalities with 1% excess (2.3851 mol/kg), which resulted in a decrease in the concentration of Mg^2+^ and Ca^2+^ to ~10^−5^ wt. % (Table 2). During the leaching of the model solution with Na_2_CO_3_, the concentration of NaCl exceeds its solubility limit, and it also starts to precipitate, which explains the decrease of Cl^−^ molality during the treatment. It should also be noted that the molality of Li^+^ remains the same during the leaching, but the weight concentration increases due to a decrease in the density and total weight of the solution.

Thermodynamic modeling was not the goal of this paper, which is why a number of simplifications were made for further modeling with Simulink. Potassium ions were included as a contribution to sodium ions because K^+^ molality is about 10 times smaller than that of Na^+^, and their behavior is similar; bromine ions were included in the mass of chlorine ions for the same reasons. Simulation in PHREEQC with Pitzer database at elevated temperatures indicated that CaCO_3_ and MgCO_3_ solubility in NaCl saturated solutions decreases with temperature (positive saturation indexes) but increases for CaMg(CO_3_)_2_ (negative saturation indexes). There are very limited experimental data on the complex interaction of magnesium with NaCl [64]. We assumed that its behavior in concentrated NaCl solution is similar to calcium and summed up their concentrations. Literature data, especially the paper [65], indicate that CaCO_3_ solubility is extremely dependent on pH and CO_2_ pressure and can differ by orders of magnitude [66,67]. There are several reported data on the phase diagram CaCO_3_-NaCl-H_2_O at room temperature, but to our best knowledge, no comprehensive study on the solubility of CaCO_3_ in saturated NaCl solutions at elevated temperatures was reported. However, according to [67,68,69], the solubility of CaCO_3_ in a solution of 3 mol/kg NaCl at 25 °C (~0.6 mmol/kg) was lower than in 3.3 mol/kg NaCl at 60 °C (~0.7 mmol/kg) which may indicate the increase of CaCO_3_ solubility in saturated NaCl solutions. Even if CaCO_3_ and MgCO_3_ precipitate, their amount in the solution was negligible, which is why no special attention was given to the fouling of the membranes by precipitated hardness salts in the Simulink simulation. Such assumptions resulted in a solution saturated with NaCl at room temperature (~26.4 wt. %), with Li^+^ concentration 0.01146 wt. % (or 0.138 g/L) and summarized Ca^2+^ + Mg^2+^ content ~3·10^−5^ wt. %, which was used as a make-up flow in Simulink calculations (Table 2).

### 3.3. Simulation of MD and ME Performance

**Figure 7:**
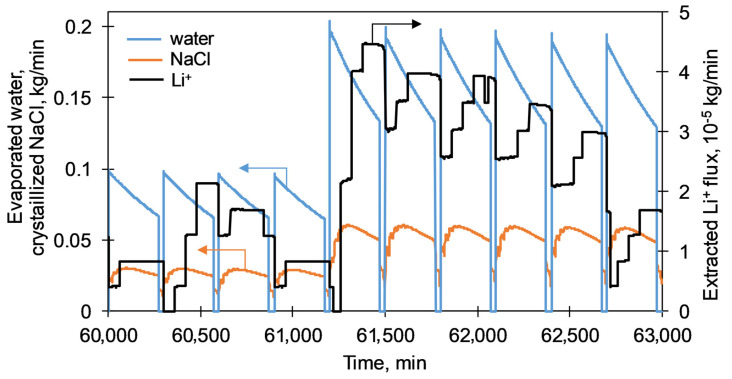
Change in time of amount of evaporated water, precipitated NaCl and recovered Li^+^. Simulation conditions: T_f_ = 60 °C, Rt = 4 h, RR = 99%, MPT = 50%, S = 2.5 m^2^, CF = 20, ER(Li^+^) = 90%.

Based on primary simulations, the area of the membrane module was set as 2.5 m^2^; the temperature of the feed solution *T_f_* was used as 60 °C. Figure 7 shows the typical results of system performance in terms of amount of evaporated water, precipitated salts, and recovered lithium in time. The lithium concentration was already reached 2.3 g/kg, which is attributed to a concentration factor (CF) of 20, and the system was maintaining the mass-balance in the circulated liquid loop in response to the deterioration of membrane performance due to its scaling/fouling. The decline of the amount of evaporated water (see blue line on Figure 7) in time can be seen during each single membrane run of 4.5 h followed by the periodic stopover for the membrane washing (0.5 h) resulting in the recovery of membrane performance (recovery ratio RF was set as 99% here). At the time of 60,900 min from the beginning of system operation, the washed membrane demonstrated the evaporated water flux at the level of 50% from its initial value, and therefore the membrane module was replaced by the new one instead of washing since the membrane performance threshold parameter was set as 50%. Thus, at the next run starting from the time of 61,170 min, the amount of evaporated water was doubled in contrast to the previous run. Since the water evaporation affected the change of solution composition, the corresponding fluctuation of precipitated sodium chloride in the crystallizer (see orange line on Figure 7) can be noticed. In addition, the system was responded to the increasing concentration of lithium above its predefined level of 2.3 g/kg to feed a larger volume of brine solution to the membrane extractor where lithium ions (see a black line on Figure 7) were recovered with an extraction rate ER(Li^+^) of 90%. Due to the simplification of the process simulation, the solution was continuously circulated within the system even during the washing or replacement of the membrane module, which explains the continuation of salt precipitation and lithium recovery at the stopovers of membrane module operations. Since the primary focus was given on the membrane module performance, the stopover of crystallization and membrane extraction units for their maintenance rather the deterioration of their performance in time were not considered in this work; and it will be done in further investigations.

#### 3.3.1. Effect of Membrane Module Performance

As mentioned earlier, the goal of this work was to consider the effect of membrane distillation performance on the operation of the whole integrated system. Since the membrane was continuously contacted with the nearly saturated saline solution having about the same composition of NaCl, it was assumed that the membrane module demonstrated the same decline of the performance in time regardless of the regime of operation and a prior number of washings. To evaluate the effect of membrane performance, the following parameters were varied: (i) run time before membrane washing RT as 4.5, 10, or 20 h, (ii) recovery ratio of membrane performance after the washing RR as 99, 97, or 95%, (iii) membrane performance threshold MPT, when the membrane module was replaced by the new one, as 50, 40, or 30%. Build-up was defined as a time from the beginning of operation until the specified lithium content was achieved and the extraction began. Table 5 shows how the run time RT and recovery rate RR effect the time of lithium concentration build-up, number of replaced membrane modules, number of membrane washings, amount of recovered lithium, crystallized NaCl, and evaporated water. These are results of the simulation of two months of operation with the lithium concentration in the circulated solution at about 2.3 g/kg. The quite small surface area of the membrane module (2.6 m^2^) was taken to consider more frequent change within two months, which resulted in high values of build-up time of lithium concentration in the liquid loop of ~140 kg having a continuous make-up flow of fresh saline solution.

As expected, the efficiency of membrane module washing and the presence of irreversible membrane fouling became a noticeable parameter during the long-term operation. Considering the case of a 4.5 h run of the membrane module, the drop of recovery ratio RR after the washing from 99% down to 95% resulted in the dramatic increase of membrane module replacement from 4 up to 21 during two months of operation (see Table 5). The prolongation of membrane operation before the washing from 4.5 up to 20 h can significantly reduce the required number of modules (S = 2.5 m^2^) – 1 and 5 at recovery ratios of 99 and 95%, respectively. However, the longer time of operation is attributed to the lower mass-transfer characteristics since the membrane module would undergo the drop of its performance within one single run for 82% at 20 h, 57% at 10 h, and 27% at 4.5 h. As a result, the drop in the amount of evaporated water within two months from ~9100 down to ~6000 kg would also lead to the decrease of recovered lithium from ~1.4 down to ~0.9 kg. However, such drawbacks seem less critical compared with the necessity of a larger amount of modules (4–21 vs. 1–5) and more frequent washing of membrane modules (288 vs. 70). It should be pointed out that the recovery ratio RR has a great impact only on the number of replaced membrane modules, while other outcome parameters mainly depended on the run time before washing Rt. The build-up time can be further reduced with the improvement of process efficiency by avoiding the system stopover during the washing step by the installation of a second membrane module in parallel, which can be operated while the first one is being washed.

**Figure 8:**
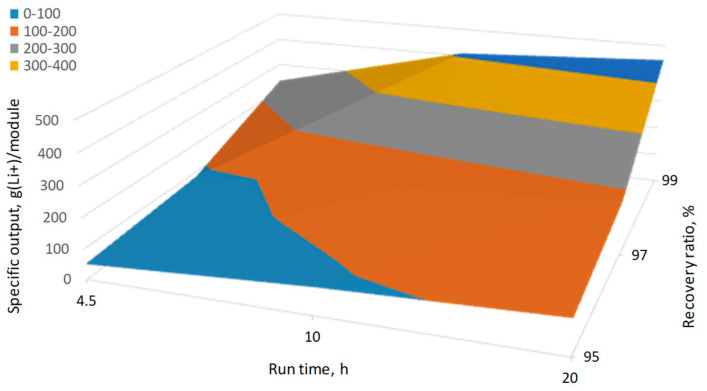
Effect of run time Rt and recovery ratio RR on lithium recovery per membrane module. Simulation conditions: T_f_ = 60 °C, Rt = 4 h, RR = 99%, MPT = 50%, S = 2.5 m^2^, CF = 20, ER(Li^+^) = 90%.

To sum up, it can be concluded that the short-term operation of the membrane module before washing can be more favorable over the longer-term one only if it allowed to significantly reduce the irreversible fouling (see data for Rt = 4.5 h/RR = 99% and Rt = 20 h/RR = 95% in Table 5). Otherwise, it is rather difficult to expect that a higher yield of extracted lithium could compensate the dramatic increase of required membrane modules. Figure 8 summarizes the extracted lithium per used membrane module at different run time Rt and recovery ratio RR. As already shown in Table 5 and discussed above, the specific lithium recovery per membrane module used is mainly determined by the recovery ratio RR, which defines the lifetime of the membrane module, rather than the run time of the module before washing Rt. The further optimization of the membrane module used can be achieved by the increase of their lifetime before replacement. As can be seen from Table 6, the drop in the membrane performance threshold MPT from 50 down to 30% for the worst-case scenario considered in this work (Rt = 4.5 h, RR of 95%) can decrease the number of replaced membrane modules from 21 down to 12 resulted in the increase of specific recovery rate from 67 up to 107 g(Li^+^)/module. In addition, we have also used the literature data on the concentration of nearly saturated brine solution by using a different configuration of membrane distillation for process simulation (Table 7). To our best knowledge, the simulation made based on the different membrane performance revealed the highest specific output was 2200 g(Li^+^) per module used (surface area of 2.5 m^2^ was fixed) for the integrated system based on the direct contact membrane distillation and multi-stage crystallization system [53] (see Table 6). In other words, the best reported in the literature configuration would allow for recovering 880 g(Li^+^) per 1 m^2^ of the membrane in the module. Taking into account the fact that the recovery of 0.9-1.4 kg of Li^+^ was associated with the precipitation of about 2.1–3.3 tons of NaCl in the crystallizer (see Table 5), the potential loss of lithium as a result of so-crystallization shall also be considered in the further study.

The energy and cost evaluations were out of the scope of this study; however, it should be pointed out that 1/3 of hot solution at 60 °C after the membrane distillation unit was fed to the crystallizer to be cooled down to 15 °C. Variation of different system parameters did not noticeably change the mass flow-rate of saline solution that went to the crystallizer (6 kg/min), which means that about 259 tons of saline solution per month had to be cooled down and then heated up. Bearing this in mind, a very critical parameter for this application would be not only the cost and robust performance of the membrane modules but also the effective heat recuperation, which will be complicated by the fact that the saline solution is near saturation or oversaturation with regard to its temperature. As a result, not all developments and achievements proposed and used for effective heat recuperation in the membrane distillation of low or moderate saline solutions would be applicable in the membrane crystallization process.

#### 3.3.2. Effect of Lithium Recovery and Concentration

As discussed above, the efficiency of the membrane extraction process is based on the lithium concentration in the feed solution, which should be 0.7 g/L and preferably above 2.0 g/L. Therefore, we have considered three case scenarios, when the initial saline solution was concentrated by the factor of 5, 20, and 50 to achieve Li^+^ concentration in the liquid loop of 0.6, 2.3, or 6.0 g/kg, respectively. Table 8 presents the data for the initial build-up step and followed by two months of operation when the desired lithium concentration was reached and maintained at the same level. As can be seen, Li^+^ presence in the 140 kg of the solution with the continuous make-up flow can be increased by a factor of 5 within 92 h by using one membrane module. However, one fold change of concentration factor CF from 5 up to 50 required 13.3 times longer time of operation (92 vs. 1227 h) and three replacements of membrane modules to reach lithium concentration of 6.0 g/kg. Ten times higher concentration of lithium in the circulated solution does not lead to a noticeable change of the process parameters except the dramatic reduction of the amount of solution fed to the membrane extraction unit by a factor of 6.2 (1662 vs. 269 kg). The increase of lithium recovery by 6% was also noticed, but this improvement in lithium extraction would be greater once the membrane extraction process is considered and simulated in detail. The decrease of extraction rate of lithium from 90 down to 50% did not have a noticeable impact on the recovered lithium in this simulation because this change of ER(Li^+^) parameter was compensated by a nearly double increase of the amount of solution fed to the membrane extraction unit from 650 up to 1151 kg during two months of operation (see Table 9).

The efficiency of the process can be significantly improved by the raising of lithium concentration in the geothermal brine. Table 10 shows the results of the simulation of the integrated system for two different geothermal water sources—Udachnaya diamond pipe (Li^+^ = 0.14 g/L) and Znamenskoe (Li^+^ = 0.48 g/L). The increase of lithium content in the make-up flow by a factor of 3.4 allowed for reaching the steady-state regime of operation within a much shorter time (165 vs. 740 h) and to recover ~3.3 kg of lithium within two months.

## 4. Conclusions

Lithium-rich geothermal waters are considered as an alternative source, and further concentration of lithium is required for its effective recovery. However, the high salinity of such geothermal brines including the presence of magnesium cations hinders their wide utilization. Since the concentration of brine solution would be attributed to the highest energy cost, the membrane distillation seems a promising approach since the low-grade heat (e.g., 60 °C) can be utilized. In this work, we have simulated a three-stage lithium recovery process including the brine softening by precipitation Ca^2+^/Mg^2+^ cations with sodium carbonate (calculated in PHREEQC), followed by an integrated system consisting of membrane distillation unit (water evaporation), crystallizer (NaCl precipitation), and membrane extraction (Li^+^ recovery), which was simulated in Simulink/MATLAB. Simulink/MATLAB allows for simulating the operation of different units integrated into one system in the real-time regime. The small membrane surface area of 2.5 m^2^ for the integrated system of 140 kg of saline solution was selected to accumulate a sufficient number of membrane replacements within two months of operation for the evaluation. Based on the obtained results, the following conclusions and comments can be made:

1. High robust, fouling resistance membranes are needed. The deterioration of membrane performance in time due to scaling/fouling plays a critical role in the performance of the system. For example, if the membranes undergo greater irreversible fouling resulting in the recovery ratio of water flux after its washing every 4.5 h at 95% from the previous value instead of 99%, then two months of operation would require 21 replacements of membrane modules instead of 4. The specific lithium recovery per square meter of membrane in the module is mainly determined by the recovery ratio, which defines the lifetime of the membrane module, rather than the run time of the module before washing.

2. Low cost membranes are required. The process simulation based on the experimental and literature data on the high salinity solutions with the membrane distillation revealed that the specific productivity is within the range of 9.9–880 g(Li^+^) per square meter of membranes in the module used before the replacement, which makes 0.053-4.7 kg in the form of lithium carbonate or 0.5-42 USD (lithium carbonate price—58.5 CNY/kg at China Spot on 18.02.2021). However, all direct and indirect costs must be accounted; for instance, the microfiltration, hydrophobic membrane MFFK-1 used in this study to carry out the membrane distillation experiments costs 1300 Rub/m^2^ (~17.5 USD/m^2^).

3. The increase of energy efficiency is needed. The mass-flow-rate of saline solution circulated to the crystallizer was set at its almost minimum value as 6.5 kg/min to enable its successful operation at the given parameters of the membrane distillation unit. In other words, the operation of the integrated system having 140 kg of saline solution in the loop, membrane module of 2.5 m^2^ for concentration of lithium presence from 0.11 up to 2.3 g/kg would be associated with the circulation of about of 259 tons of saline solution per month between the distillation unit (60 °C) and the crystallizer (15 °C) to a yield of up to 1.4 kg of lithium ions. Therefore, it will be critical to implement the effective heat recuperation, which will be complicated by the fact that the saline solution is near saturation or oversaturation with regard to its temperature.

4. To increase the attractiveness of the geothermal brines as an alternative lithium source, novel concentration methods of high saline solutions with high robustness to the scaling and fouling during the long-term operation are needed. For instance, the thin-film distillation coupled with membrane condenser for brine solutions concentration was recently proposed [47]. In addition, lower temperature difference between different units of the integrated system might overcome the problem of salt precipitation and enable more effective heat recuperation.

## Figures and Tables

**Figure 3:**
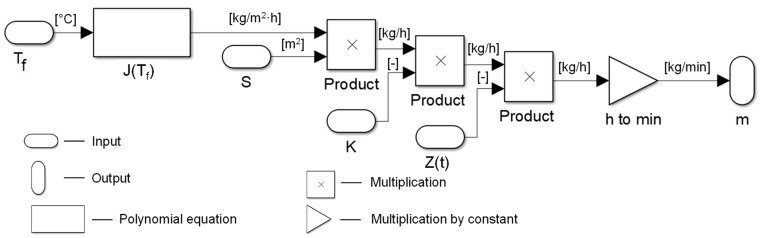
Representation of the membrane module in Simulink: *m* is the amount of water evaporated in MD module per minute [kg/min], *J*(*T_f_*) is the polynomial equation that defines the flux of distilled water as a function of the feed Table 2 [kg/m^2^·h]; *T_f_* is the feed temperature [°C]; *S* is the active surface area of one membrane module [m^2^], *K* is the number of membrane modules, *Z*(*t*) is the nonlinear function describing the deterioration of membrane performance due to scaling/fouling over time.

**Table 1:** Composition of several lithium-rich geothermal brines in Russia [25,26].

Deposit Name	Concentration, g/L Solution							
	Li^+^	Na^+^	K^+^	Mg^2+^	Ca^2+^	Cl^−^	Br^−^	Σsalts
Dagestan, Tarumovskoe	**0.2**	67.0	3.8	0.8	10.1	127.3	0.6	**210**
Sakha Republic, Udachnaya diamond pipe	**0.14**	35.6	20.3	11.2	65.5	220.0	4.8	**360**
Krasnoyarsk region, Suho-Tungusskoe	**0.22**	46.8	23.1	9.5	58.8	233.9	3.2	**375**
Krasnoyarsk region, Verkhnekostinskoe	**0.45**	50.2	19.7	11.2	81.7	271.8	5.6	**444**
Irkutsk region, Znamenskoe	**0.48**	2.4	4.3	28.5	134.3	322.5	10.6	**503**
Irkutsk region, Kovyktinskoe	**0.39**	1.9	11.7	29.0	154.0	338.9	6.3	**544**

**Table 2:** Composition of the fresh feed (make-up) solution taken for simulation.

	Li^+^	NaCl	Mg^2+^/Ca^2+^	H_2_O
**Molality, mol/kg water**	0.0227	6.129	10^−5^	55.51
**Concentration, wt. %**	0.01146	26.4	3∙10^−5^	73.59

**Table 3:** Comparison of performance drop in the membrane distillation with crystallization for NaCl saturated solution.

Process	Membrane	Temperatures, °C (Hot/Cold)	Duration of One Run, h	Flux Drop in One Run, %	Recovery Ratio, %	Source
DCMD	PP	80/50	20	30.3	82	[49]
DCMD	PVDF	65/30	4	61.8	94	[52]
DCMD	PP	85/55	42	10.7	94	[53]
DCMD	PVDF	60/15	0.6	5–15	98.6	[54]
DCMD	CF4-MP-PVDF	60/20	-	35	-	[55]
DCMD	PVDF	70–80/20	5–8.5	92–93	-	[56]
DCMD	PVDF	50–60/20–30	5–8.5	100	-	[57]
DCMD	PTFE	80/70	0.8	100	-	[58]
SMDC	PVDF	40–70/25	3.3	0–47	-	[59]
DCMD	PFDV/PAN	60/25	9	40	-	[60]
VMD	PTFE	50/vacuum	6	24	-	[61]
AGMD-MC	MFFK-1	60–80/20	2–4.5	24	99	this work

**Table 4:** Change in the composition of the geothermal brine during soda ash treatment (calculation with PHREEQC).

**.**	**Molality, mol/kg Water**									**Solution Density, kg/m^3^**
	**Li^+^**	**Na^+^**	**K^+^**	**Mg^2+^**	**Ca^2+^**	**Cl^−^**	**Br^−^**	**Carbonates**	**H_2_O**	
Before treatment	0.0227	1.746	0.5853	0.5195	1.842	7.009	0.0677		55.51	1244.7
After treatment	0.0227	5.848	0.5854	5.14∙10^−6^	6.02∙10^−6^	6.341	0.0677	0.0314	55.51	1210.0
	**Concentration, wt. %**									
Before treatment	0.01134	2.86	1.63	0.90	5.26	17.70	0.39		71.25	1244.7
After treatment	0.01146	9.68	1.64	8.98∙10^−6^	1.74·10^−5^	16.18	0.39	0.14	71.96	1210.0

**Table 5:** Effect of run time RT and recovery ratio RR on process parameters (two months of operation). Simulation conditions: T_f_ = 60 °C, MPT = 50%, S = 2.5 m^2^, CF = 20, ER(Li^+^) = 90%.

Run Time Rt, Hour	Recovery Ratio RR, %	Build-Up Time, Hours	Number of Membrane Washings	Number of Replaced Membrane Modules	Recovered Li^+^, kg	Evaporated Water, kg	Crystallized NaCl, kg
4.5	99	467	288	4	1.410	9231	3289
4.5	97	502	288	12	1.424	9138	3313
4.5	95	491	288	21	1.404	9173	3308
10	99	528	137	2	1.228	8101	2880
10	97	548	137	6	1.223	8019	2849
10	95	569	137	10	1.242	8018	2850
20	99	658	70	1	0.898	6152	2181
20	97	740	70	3	0.902	5938	2105
20	95	760	70	5	0.909	5912	2096

**Table 6:** Effect of membrane performance threshold MPT on process parameters (two months of operation). Simulation conditions: T_f_ = 60°C, Rt = 4.5 h, S = 2.5 m^2^, CF = 20, ER(Li^+^) = 90%.

Membrane Performance Threshold MPT, %	Recovery Ratio RR, %	Build-Up Time, Hours	Number of Membrane Washing	Number of Replaced Membrane Modules	Recovered Li^+^, kg	Evaporated Water, kg	Crystalized NaCl, kg	Specific Output, g(Li^+^)/Module
50	99	467	288	4	1410	9231	3289	353
40	99	528	288	3	1275	8321	3055	425
30	99	618	288	3	1176	7520	2886	392
50	95	491	288	21	1410	9173	3308	67
40	95	541	288	16	1274	8296	2952	80
30	95	612	288	12	1287	7254	2579	107

**Table 7:** Comparison of different configurations and performance of MD process reported in the literature and inthis work (two months of operation). Simulation conditions: RR = 50%, S = 2.5 m^2^, CF = 20, ER(Li^+^) = 90%.

MD Process Configuration	Temperature Mode (Hot/Cold side)	Build-Up Time, Hours	Number of Membrane Washings	Number of Replaced Membrane Modules	Recovered Li^+^, kg	Evaporated Water, kg	Crystallized NaCl, kg	Specific Output, g(Li^+^)/Module	Ref.
Hollow fiber (DCMD)	65/30	778	480	37	0.914	5836	2076	24.7	[52]
Hollow fiber (DCMD)	85/25	228	72	17	3.954	20901	7456	233	[49]
Hollow fiber (DCMD)	85/50	131	34	2	4.427	29134	10399	2214	[61]
Flat sheet (AGMD-MC)	60/20	467	288	4	1.410	9231	3290	353	This work
Flat sheet (AGMD-MC)	80/20	333	288	5	2.138	13677	4879	428	This work

**Table 8:** Effect of concentration factor CF on processes operation. Simulation conditions: T_f_ = 60 °C, Rt = 4.5 h, RR = 99%, S = 2.5 m^2^, CF = 20, MPT = 50%, ER(Li^+^) = 90%.

Concentration Factor CF	Build-Up					Two Months of Operation	
	Time, Hours	Number of Membrane Washings	Number of Replaced Membrane Modules	Evaporated Water, kg	Crystallized NaCl, kg	Recovered Li^+^, kg	Mass of Solution Fed to ME Module, kg
5	92	18	0	593	169	1.347	1662
20	467	93	1	2996	1068	1.410	650
50	1227	245	3	7801	3894	1.427	269

**Table 9:** Impact of extraction rate on membrane distillation processes (two months of operation). Simulation conditions: T_f_ = 60 °C, Rt = 4.5 h, RR = 99%, S = 2.5 m^2^, MPT = 50%.

Extraction Rate ER(Li^+^), %	Recovered Li^+^, kg	Mass of Solution Fed to ME Module, kg	Evaporated Water, kg	Crystallized NaCl, kg
90	1.410	650	9231	3289
70	1.406	831	9237	3290
50	1.397	1151	9237	3290

**Table 10:** Results for various deposits. Simulation conditions: T_f_ = 60°C, Rt = 20 h, RR = 97%, S = 2.5 m^2^, C_Li_^+^ = 2.3 g/kg, MPT = 50%, ER(Li^+^) = 90%.

Deposit Name	Build-Up		Two Months of Operation				
	Number of Replaced Membrane Modules	Build-Up Time, Hours	Number of Replaced Membrane Modules	Recovered Li^+^, kg	Evaporated Water, kg	Crystallized NaCl, kg	Specific Output g(Li^+^)/Module
Sakha Republic, Udachnaya diamond pipe	1	740	3	0.902	5938	2105	300
Irkutsk region, Znamenskoe	0	165	3	3.270	5989	2347	1215

## Supplementary Materials

The following are available online at https://www.mdpi.com/2077-0375/11/3/175/s1, Supplementary S1: PHREEQC code for conversion the concentration from g/L to molality; Supplementary S2: PHREEQC code for simulation of CaCO_3_ and MgCO_3_ precipitation by Na_2_CO_3_. Boron was added with Na_2_CO_3_ in trace amounts to use it as a marker to draw a graph; Figure S1: Overview of the model in Simulink; Figure S2: Heater system in terms of Simulink; Figure S3: Crystallizer system in terms of Simulink; Figure S4: Crystallizer subsystem in terms of Simulink; Figure S5: Extractor system in terms of Simulink; Figure S6: Extractor subsystem in terms of Simulink; Figure S7: Membrane module system in terms of Simulink. Figure S8: Membrane fouling simulation system in terms of Simulink; Figure S9: Make-up flow system in terms of Simulink; Figure S10: Second heater subsystem in terms of Simulink; Figure S11: Subsystem for automatic calculation of the total membrane surface area in terms of Simulink.

## Nomenclature

*Acronyms*	
AGMD-MC	air–gap membrane distillation with membrane condenser
CF	concentration factor
CF4-MP-PVDF	CF4 plasma modified micro-pillared PVDF membrane
DCMD	direct contact membrane distillation
ER(Li^+^)	Li^+^ extraction rate for the ME process, %
EVAL	ethylene–vinyl alcohol
MD	membrane distillation
ME	membrane extraction
MPT	membrane performance threshold, %
PAN	polyacrylonitrile
PEEK	poly(ether-ether-ketone)
PID	proportional–integral–derivative
PP	polypropylene
PTFE	poly(tetrafluoroethylene)
PVDF	poly(vinylidene fluoride)
RR	recovery ratio, %
Rt	run time before membrane washing
SIT	specific ion interaction theory
*Symbols*	
m	total distilled water flux in the modeled system, g
J	permeate flux (flux of distilled water), kg/m^2^∙h
T_f_	feed temperature, °C
J(T_f_)	polynomial equation that defines the flux of distilled water as a function of the feed temperature, kg/m^2^∙h
S	active surface area, m^2^
K	number of membrane modules
Q	make-up flow, kg/min
Q_max_	maximum make-up flow, kg/min
V	volume, l
V_current_	current volume, l
T_c_	coolant temperature, °C
Z(t)	nonlinear function describing membrane fouling over time
*Subscripts*	
c	cooling water or coolant
f	feed
t	time
