# Supplementary material for: Operation of Three-Stage Process of Lithium Recovery from Geothermal Brine: Simulation
Source: Membranes (Basel). 2021 Feb 28;11(3):175. doi: 10.3390/membranes11030175 (PMC8001782; doi:10.3390/membranes11030175)
Supplement: Supplementary file 1 [file membranes-11-00175-s001.pdf]

membranes

Supplementary materials

Appendix S1. PHREEQC code for conversion the concentration from g/l to molality.

```
# Concentration conversion, brine from Udachnaya pipe (data from Ryabtsev)
SOLUTION 1 Udachnaya
    temp      25
    pH        7
    pe        4
    redox     pe
    units     g/l
    density   1.24465
    Br        4.8
    Ca        65.5
    Cl        220 charge
    K         20.3
    Li        0.14
    Mg        11.2
    Na        35.6
    -water    1 # kg
```

Appendix S2. PHREEQC code for simulation of $CaCO_3$ and $MgCO_3$ precipitation by Na_2CO_3. Boron was added with Na_2CO_3 in trace amounts to use it as a marker to draw a graph.

```
# Precipitation of CaCO3+MgCO3, Udachnaya pipe (data from Ryabtsev)

SOLUTION 1 Udachnaya
    temp      25
    pH        7
    pe        4
    redox     pe
    units     mol/kgw
Br            6.772e-02
Ca            1.842e+00
Cl            7.009e+00 charge
K             5.853e-01
Li            2.274e-02
Mg            5.195e-01
Na            1.746e+00
        -water    1 # kg

Reaction
B 1e-9
CO3-2 1
```

```
Na 2
#2.385115 moles 50 steps
3 moles 50 steps
EQUILIBRIUM_PHASES 1
    halite      0 00000
    CO2(g)      -3.4 100000
    calcite 0 0
    Dolomite            0 0
SELECTED_OUTPUT 1
    -file                       udachnaya2.sel
    -totals                     Ca   Mg
USER_GRAPH 1
    -headings                   Ca_tot Ca Mg Cl
    -axis_titles                "Added Na2CO3, mol/kg water" "Ca and Mg in solution, mol/kg water" "Cl in solution, mol/kg
water"
    -axis_scale x_axis          auto 3.1 auto auto
    -axis_scale y_axis          1e-07 2 auto auto log
    -axis_scale sy_axis         auto auto auto auto
    -initial_solutions          false
    -connect_simulations        true
    -plot_concentration_vs      x
  -start
 10 x=tot("B")*1e9
 30 y1=tot("Ca")
 40 y2=tot("Mg")
 50 y3=tot("Cl")
# 60 y4=tot("Na")
100 graph_x x
200 graph_y y1 y2
300 graph_sy y3
  -end
    -active                     true
USER_GRAPH 2
    -headings                   Ca_tot Ca Mg Cl
    -axis_titles                "Added Na2CO3, mol/kg water" "Ca and Mg in solution, mol/kg water" "Cl in solution, mol/kg
water"
    -axis_scale x_axis          auto 3.1 auto auto
    -axis_scale y_axis          1e-07 2 auto auto
    -axis_scale sy_axis         auto auto auto auto
    -initial_solutions          false
    -connect_simulations        true
    -plot_concentration_vs      x
```

```
  -start
 10 x=tot("B")*1e9
 30 y1=tot("Ca")
 40 y2=tot("Mg")
 50 y3=tot("Cl")
# 60 y4=tot("Na")
100 graph_x x
200 graph_y y1 y2
300 graph_sy y3
  -end
    -active                    true
end
```

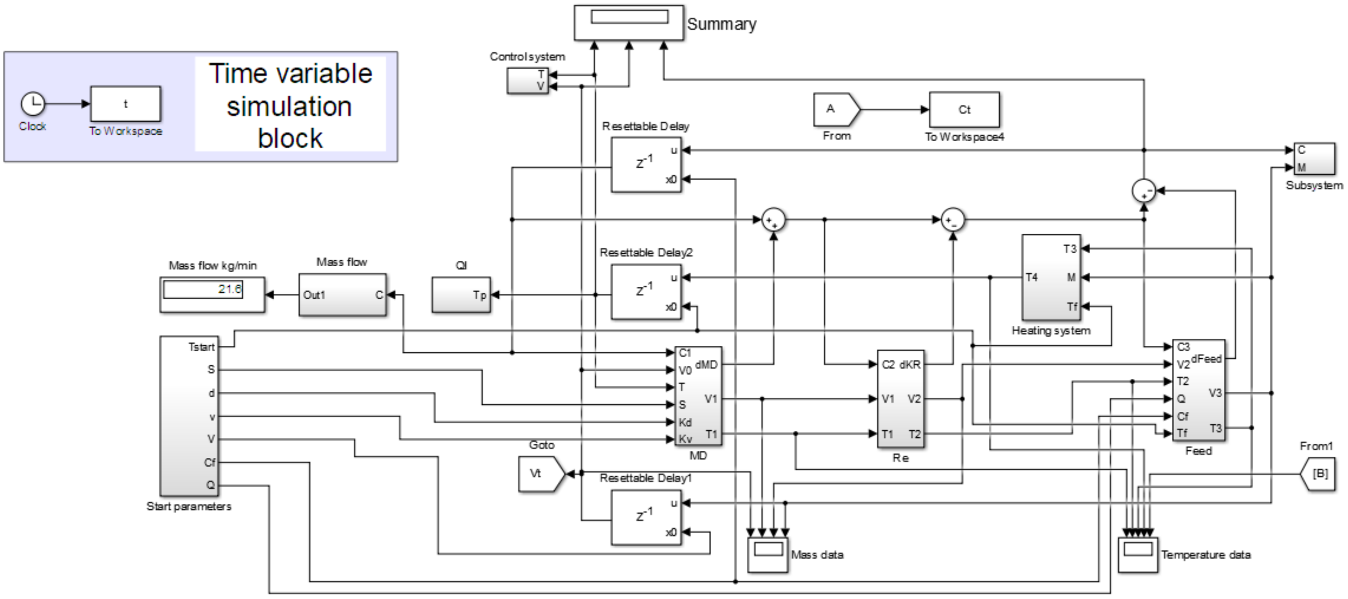

Figure S1. Overview of the model in Simulink.

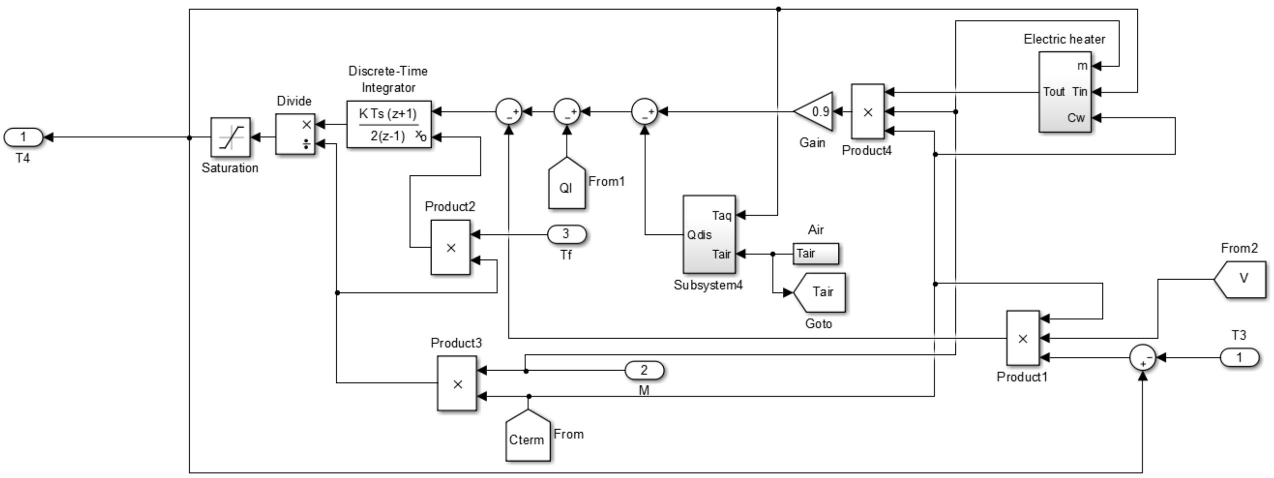

Figure S2. Heater system in terms of Simulink.

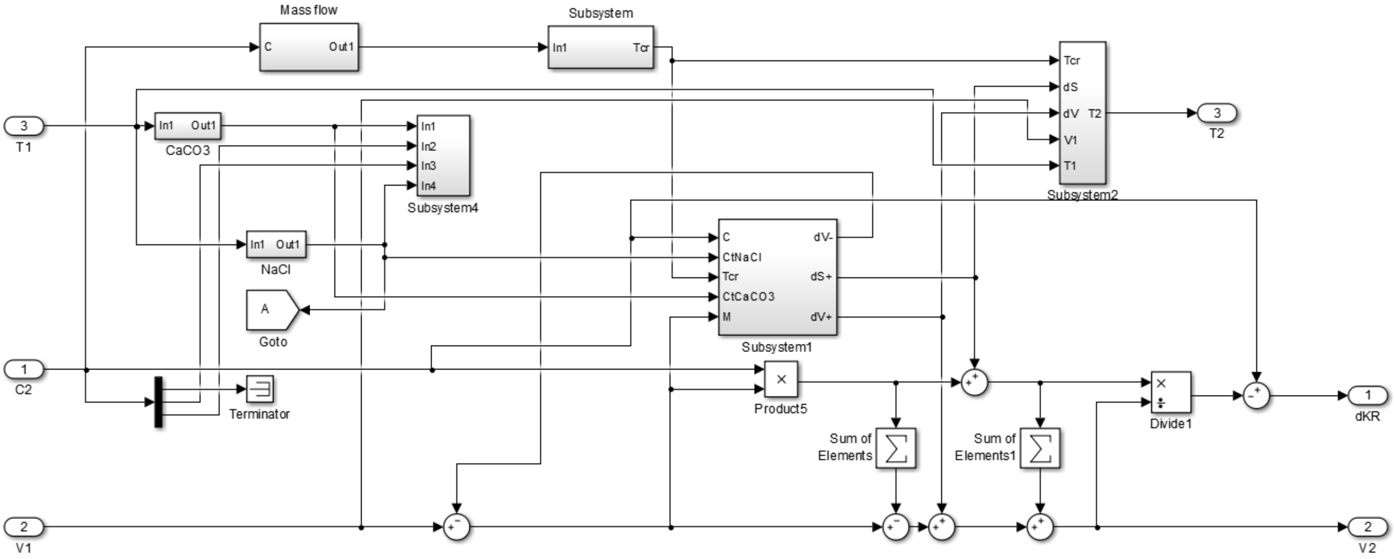

Figure S3. Crystallizer system in terms of Simulink.

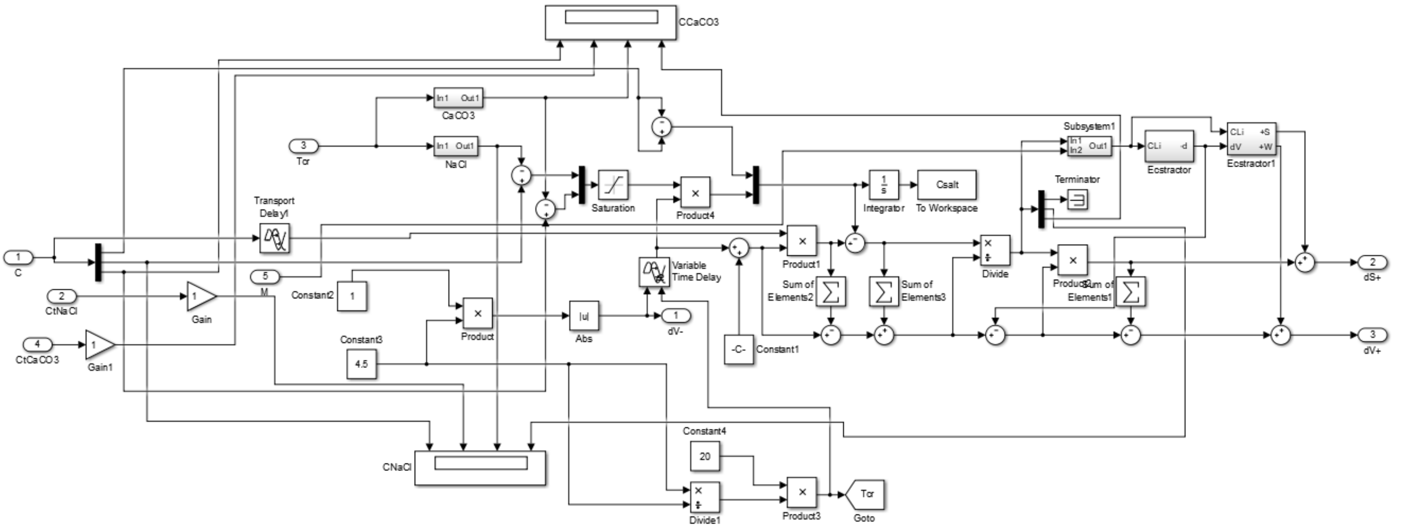

Figure S4. Crystallizer subsystem in terms of Simulink.

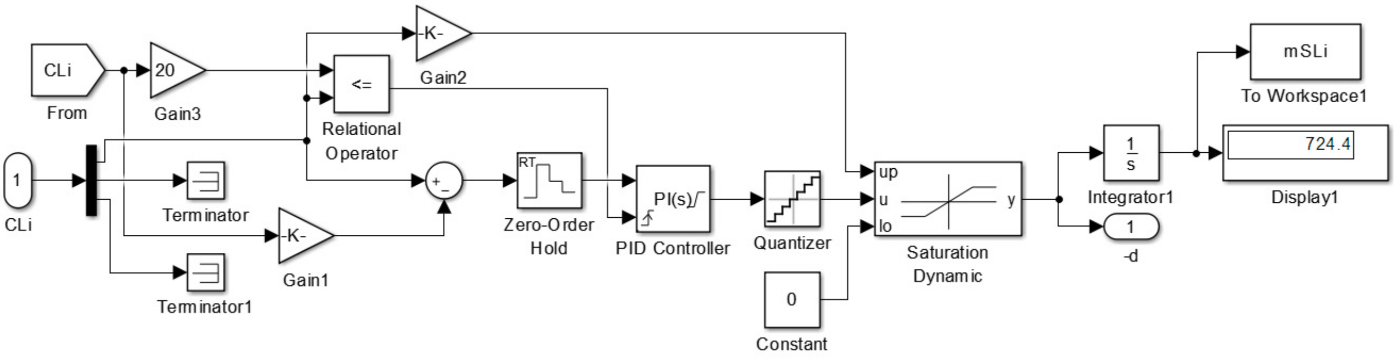

Figure S5. Extractor system in terms of Simulink.

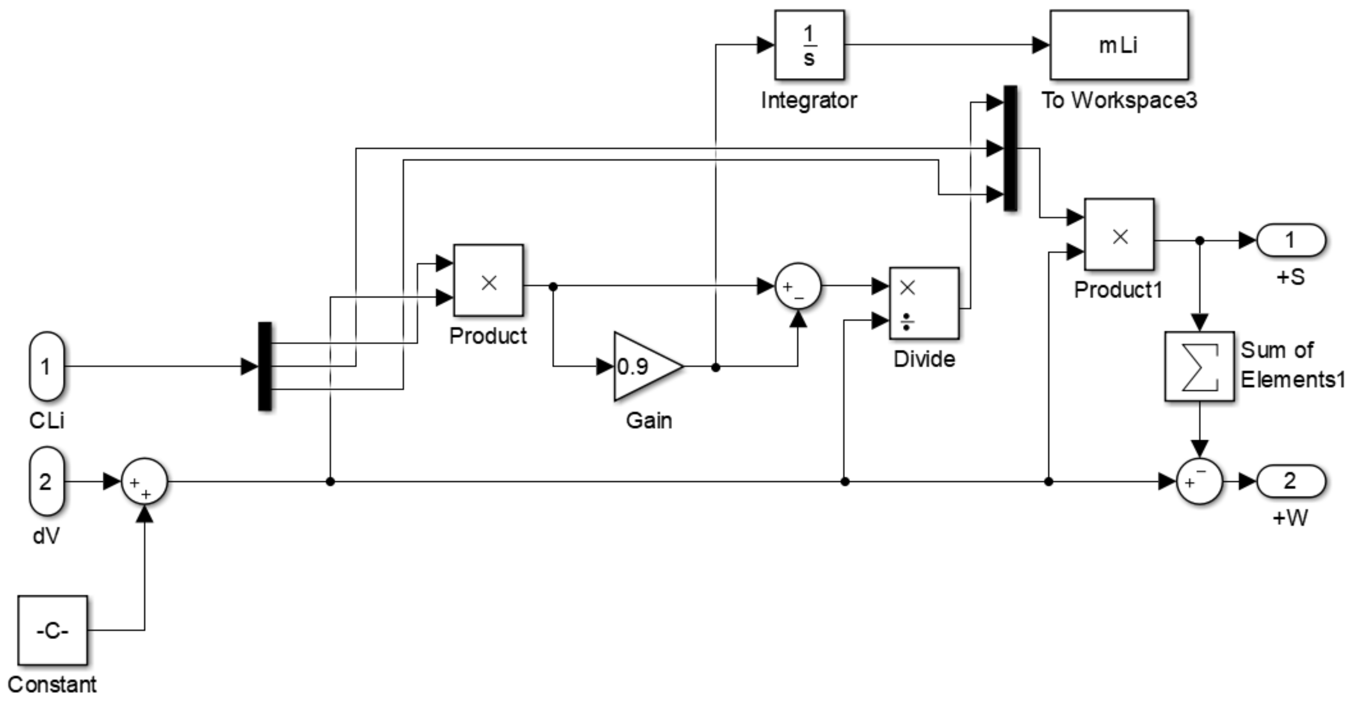

Figure S6. Extractor subsystem in terms of Simulink.

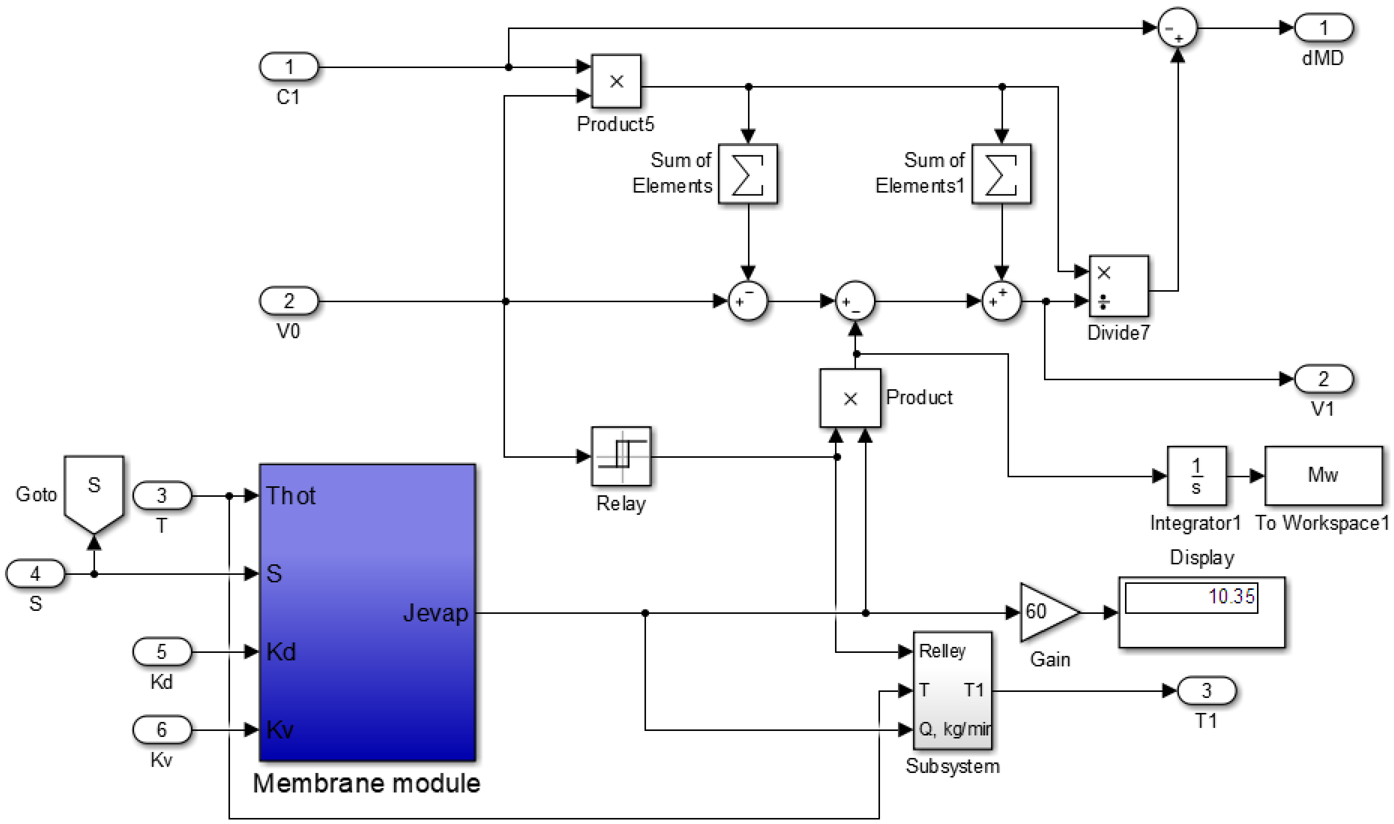

Figure S7. Membrane module system in terms of Simulink.

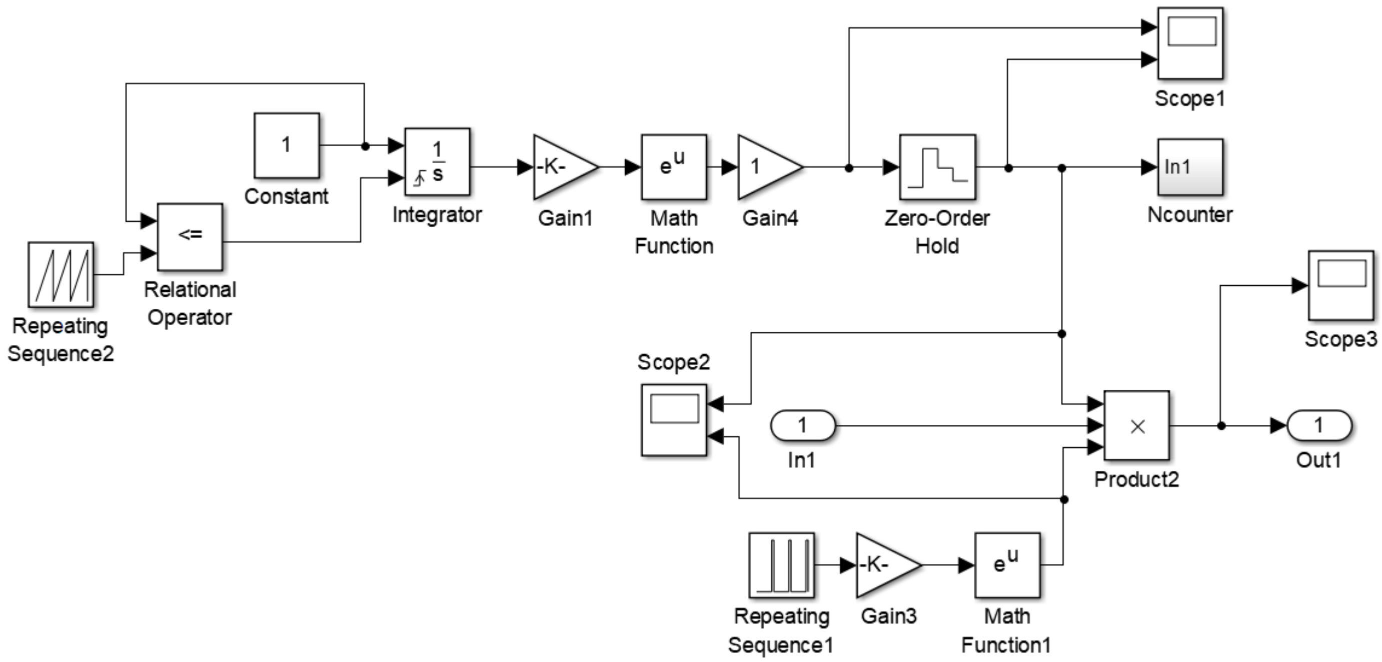

Figure S8. Membrane fouling simulation system in terms of Simulink.

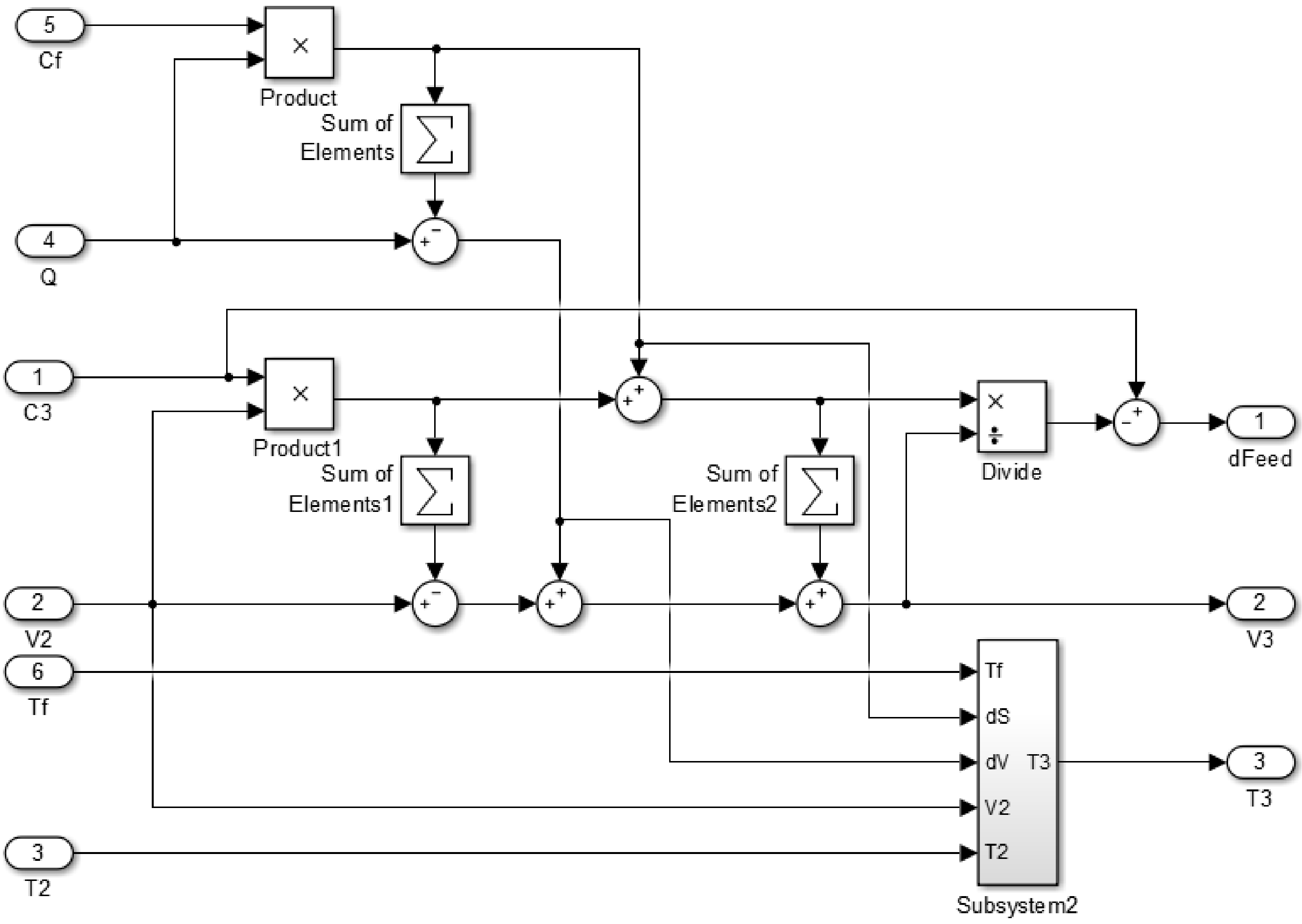

Figure S9. Make-up flow system in terms of Simulink.

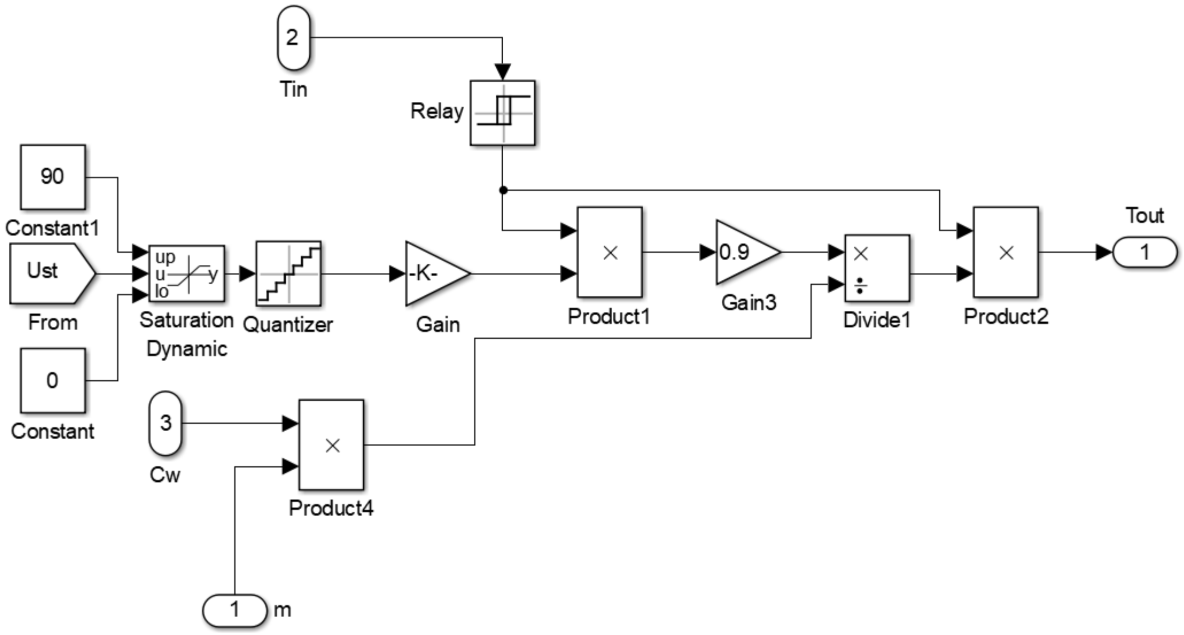

Figure S10. Second heater subsystem in terms of Simulink.

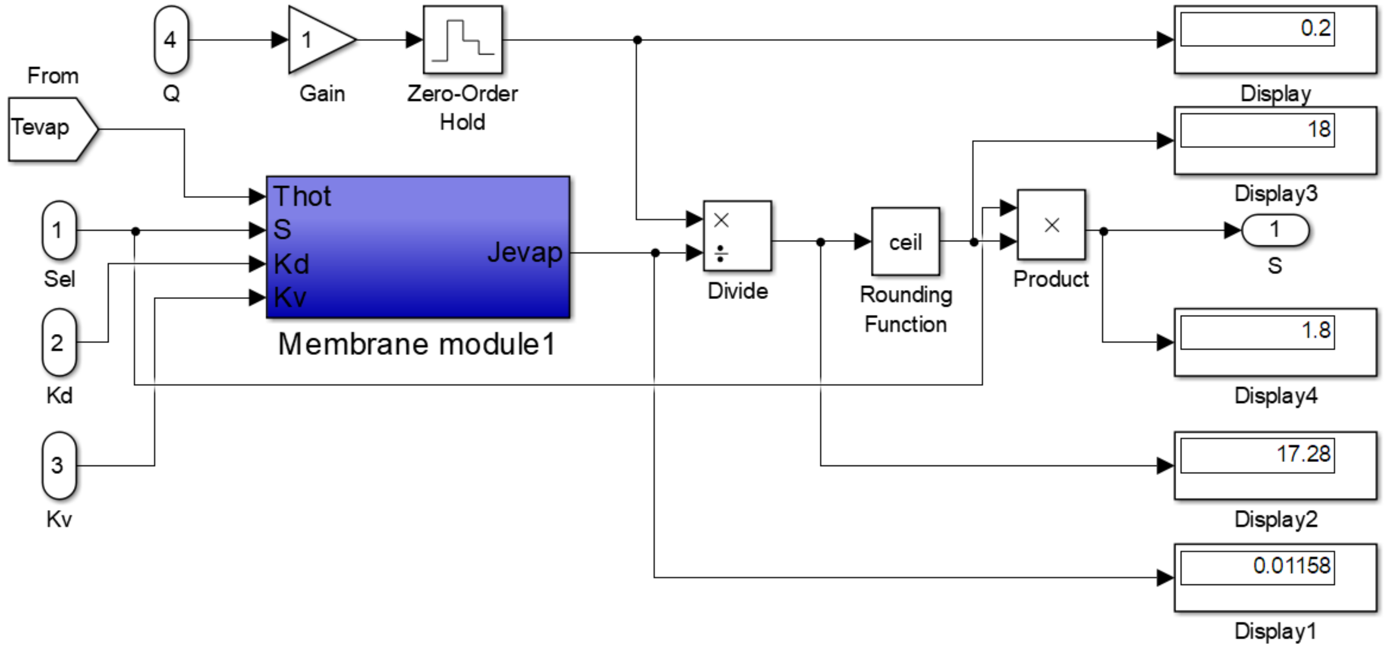

Figure S11. Subsystem for automatic calculation of the total membrane surface area in terms of Simulink.
